# Combinatorial BTK and MALT1 inhibition augments killing of CD79 mutant diffuse large B cell lymphoma

**DOI:** 10.18632/oncotarget.6273

**Published:** 2015-11-02

**Authors:** Daniel Nagel, Miriam Bognar, Andrea C. Eitelhuber, Kerstin Kutzner, Michelle Vincendeau, Daniel Krappmann

**Affiliations:** ^1^ Research Unit Cellular Signal Integration, Institute of Molecular Toxicology and Pharmacology, Helmholtz Zentrum München - German Research Center for Environmental Health, Neuherberg, Germany

**Keywords:** lymphoma therapy, combination therapy, DLBCL, BTK, MALT1

## Abstract

Survival of activated B cell-subtype (ABC) of diffuse large B cell lymphoma (DLBCL) is driven by chronic B cell receptor (BCR) signaling that activates the canonical NF-κB pathway. Inhibition of BTK by Ibrutinib has been shown to kill ABC DLBCL cells that carry activating mutations in the BCR adaptor CD79. However, mutations in BTK or in downstream components such as CARMA1/CARD11 can render lymphomas Ibrutinib resistant. Therefore, we assessed here the simultaneous inhibition of BTK and the protease MALT1 that acts downstream of CARMA1 and is essential for ABC DLBCL tumor growth. We show that in CD79 mutant cells BTK is a crucial upstream regulator of MALT1, but dispensable in CARMA1 mutant ABC DLBCL. Combined inhibition of BTK by Ibrutinib and MALT1 by S-Mepazine additively impaired MALT1 cleavage activity and expression of NF-κB pro-survival factors. Thereby, combinatorial Ibrutinib and S-Mepazine treatment enhanced killing of CD79 mutant ABC DLBCL cells. Moreover, while expression of oncogenic CARMA1 in CD79 mutant cells conferred Ibrutinib resistance, double mutant cells were still sensitive to MALT1 inhibition by S-Mepazine. Thus, based on the genetic background combinatorial BTK and MALT1 inhibition may improve effectiveness of therapeutic treatment and reduce the chances for the development of drug resistances.

## INTRODUCTION

Activated B cell-type (ABC) of diffuse-large B cell lymphoma (DLBCL) represents one of the most aggressive lymphoma entities. The high incidence of refractory and relapsed cases and the 3-year survival rate of ~ 40 % reflect the critical need for more effective therapeutic approaches. Survival of ABC DLBCL relies on chronic activation of the B cell receptor (BCR) signaling that drives activation of the canonical NF-κB pathway [1, 2]. Congruently, inactivation of BCR components or downstream signaling proteins by knock-down or pharmacological inhibition is highly toxic to ABC DLBCL [3, 4]. Further, somatic oncogenic mutations in signaling mediators that connect BCR signaling to NF-κB are frequent in ABC DLBCL patients. Activating mutations are found in the BCR proximal adaptor CD79A or CD79B (~21%) or in the scaffold protein CARMA1/CARD11 (~10%) [4, 5]. Thus, small molecule targeting of the BCR-NF-κB signaling axis represents a promising strategy to treat this highly malignant lymphoma subtype.

The tyrosine kinase BTK (Bruton's tyrosine kinase) is a key enzyme for B lymphocyte activation by linking proximal BCR signaling to various downstream pathways including AKT, calcium release, MAP kinases and NF-κB activation [6]. BTK is critical for survival and proliferation of various B cell malignancies, such as chronic lymphocytic leukemia (CLL) and mantle cell lymphoma (MCL) [6–8]. Good overall response rates in clinical trials have recently prompted FDA breakthrough approval of the irreversible BTK inhibitor Ibrutinib (PCI-32765) for the treatment of relapsed MCL and CLL [7, 9]. In addition, BTK is required for survival of CD79 mutated ABC DLBCL cells where it primarily regulates canonical NF-κB signaling [4, 10]. A first clinical trial demonstrated partial or complete responses in relapsed/refractory ABC DLBCL patients that rely on chronic BCR signaling [11]. Overall, BTK inhibition by Ibrutinib monotherapy seems to be able to control progression but is not not sufficient to fully eradicate many lymphomas [12]. Further, the appearance of Ibrutinib resistances in CLL and MCL underscores that inhibition of BCR signaling by targeting BTK alone may not be sufficient to achieve life-long responses [13–15]

The protease MALT1 acts downstream of CARMA1 in pathological BCR signaling in ABC DLBCL [3]. Congruently, MALT1 proteolytic activity is constitutively turned on in ABC DLBCL cells and promotes optimal anti-apoptotic NF-κB activation [16, 17]. Two classes of small molecule MALT1 inhibitors were recently identified that showed good preclinical responses in ABC DLBCL [18, 19]. With Mepazine and Thioridazine, two first generation anti-psychotic drugs from the class of phenothiazines have been identified as allosteric MALT1 inhibitors that kill selectively ABC DLBCL cells carrying oncogenic CD79 or CARMA1 mutations [19]. Structure-activity analyses demonstrated that the S-enantiomer of Mepazine is the most potent MALT1 inhibitor of this class of compounds [20].

Here, we determined in how far BTK inhibition by Ibrutinib is affecting MALT1 activity in ABC DLBCL cells that carry oncogenic mutations in either CD79 or CARMA1. By using defined ABC DLBCL cell lines, we evaluated the advantages of combinatorial treatment with the irreversible BTK inhibitor Ibrutinib and the allosteric MALT1 inhibitor S-Mepazine.

## RESULTS

### BTK inhibitor Ibrutinib augments MALT1 inhibition by S-Mepazine in CD79 mutant ABC DLBCL

To determine responses to Ibrutinib (PCI-32765) alone as well as Ibrutinib/S-Mepazine co-treatment on ABC DLBCL cells, we used a set of four well-characterized cell lines with distinct genetic lesions in the BCR signaling pathway. Whereas OCI-Ly3 cells express an oncogenic CARMA1 coiled-coil mutant (L244P) [5], HBL1, TMD8 and OCI-Ly10 cells carry upstream activating mutations in the ITAMs of the BCR adaptors CD79B (Y196F/HBL1 and Y196H/TMD8) or CD79A (Δ191-208/OCI-Ly10) [4]. The very frequent MYD88 mutation L265P is present in all four DLBCL cell lines [21]. Whereas all cells are characterized by constitutive MALT1 activity, only CD79 mutant cells require BTK expression [4, 16, 17].

**Figure 1:**
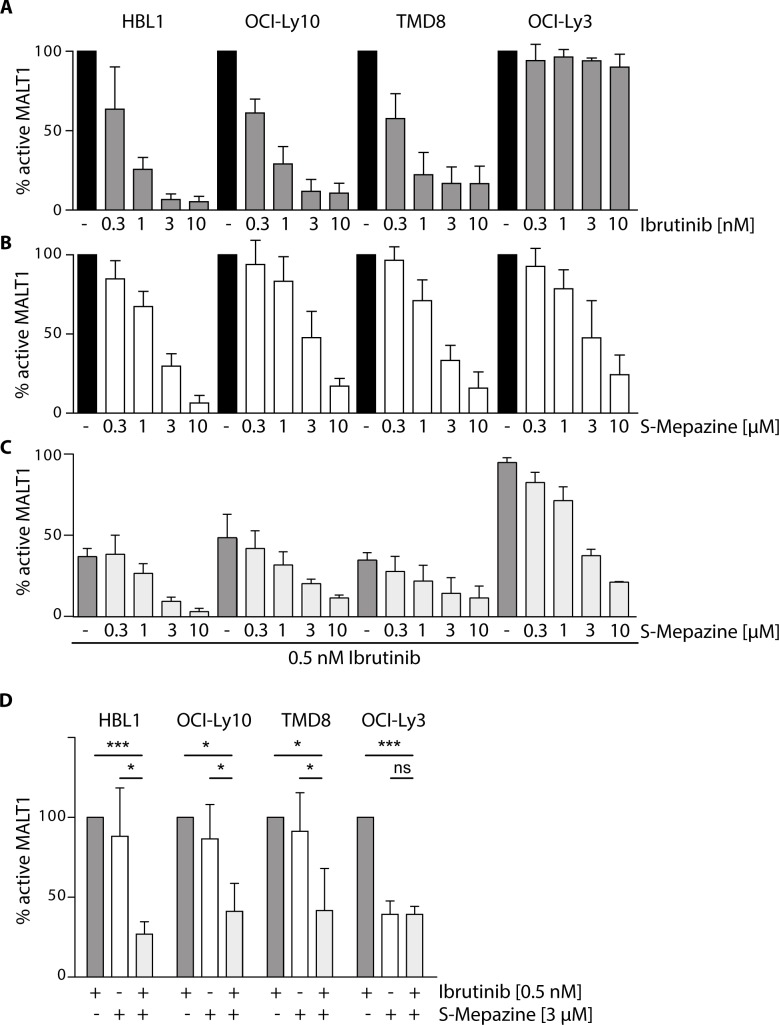
Additive effects on MALT1 activity by Ibrutinib and S-Mepazine co-treatment in CD79 mutant cells MALT1 activity was analyzed via ELASA in different ABC DLBCL cell lines (5 × 10^5^cells /ml) after 18 h treatment with either Ibrutinib **A.** or S-Mepazine **B.** alone or in combination **C.**. **A.** Ibrutinib treatment led to a dose-dependent decrease (0.3 - 10 nM) of MALT1 activity in HBL1, OCI-Ly10 and TMD8 cells, whereas MALT1 activity in OCI-Ly3 cells was not affected. **B.** S-Mepazine elicited a dose-dependent inhibition (0.3 - 10 μM) of MALT1 with a 50 % inhibition below 3 μM. **C.** Combinatorial Ibrutinib (0.5 nM) and S-Mepazine (0.3 - 10 μM) treatment resulted in augmented reduction of MALT1 activity in the ABC DLBCL cell lines HBL1, OCI-Ly10 and TMD8, but not in OCI-Ly3. **D.** Combinatorial treatment of Ibrutinib and S-Mepazine significantly reduced MALT1 activity. MALT1 activity in response to Ibrutinib was set to 100% and compared to S-Mepazine alone or S-Mepazine/Ibrutinib treatment. All data show the mean of at least three independent experiments ± SD. Significance was calculated by Student's t-test (*p ≤ 0.05; **p ≤ 0.01; ***p ≤ 0.001).

We first determined if inhibition of BTK activity by Ibrutinib affects activity of the downstream protease MALT1. For this, we directly measured MALT1 activity using chemical activity-based probes (ABP) that selectively label the active MALT1 in cellular extracts [22]. Biotinylated MALT1-ABP was used to label active MALT1 in ABC DLBCL cells and detection was done on streptavidin-coated plates by an enzyme linked activity-sorbent assay (ELASA) (Figure 1). A single treatment with increasing concentrations of Ibrutinib (0.3-10 nM) for 18 h reduced MALT1 activity in a dose dependent manner in CD79 mutant cells HBL1, OCI-Ly10 and TMD8 cells (Figure 1A). However, MALT1 activity was not affected in CARMA1 mutant OCI-Ly3 cells. In contrast, the allosteric MALT1 inhibitor S-Mepazine inhibited MALT1 in all ABC DLBCL cell lines at concentrations between 0.3-10 μM (Figure 1B). To test combinatorial effects, we incubated the cells with 0.5 nM Ibrutinib to achieve approximately 50 - 60% reduction of MALT1 activity and added increasing concentrations of S-Mepazine (Figure 1C). Combinatorial treatment of Ibrutinib and S-Mepazine augmented MALT1 inhibition in Ibrutinib responsive cells. Clearly, co-administration of Ibrutinib (0.5 nM) and S-Mepazine (3 μM) resulted in a significant reduction of MALT1 activity when compared to single agent treatment in CD79B or CD79A mutant HBL1, TMD8 or OCI-Ly10 cells, but not in Ibrutinib resistant CARMA1 mutant OCI-Ly3 cells (Figure 1D).

**Figure 2:**
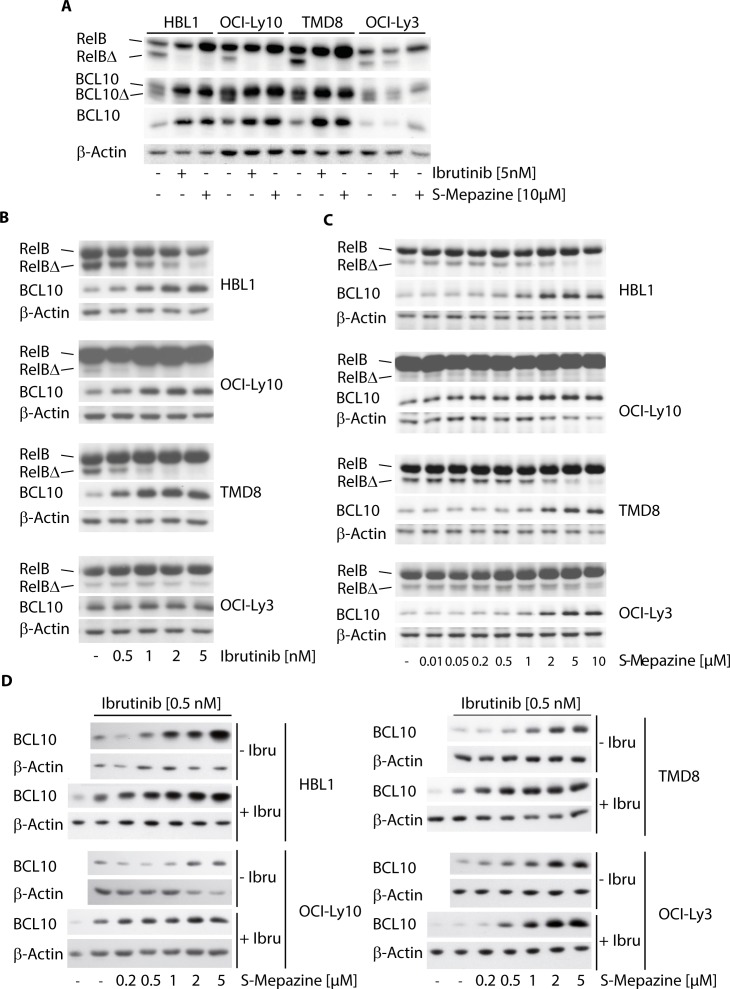
Additive effects on MALT1 substrate cleavage by Ibrutinib and S-Mepazine co-treatment in CD79 mutant cells **A.** Cleavage of MALT1 substrates RelB and BCL10 was analyzed after treatment of HBL1, OCI-Ly10, TMD8 and OCI-Ly3 cells (2.5 × 10^5^/ml) with Ibrutinib (5 nM) or S-Mepazine (10 μM) for 18 h. Cleavage products for RelB (RelBΔ) and BCL10 (BCL10Δ; antibody SC H197) were detected by Western Blot. BCL10 antibody Abcam EP606Y (lower BCL10 panel) exclusively recognizes accumulation of BCL10 full-length proteins. **B and C.** Cleavage of MALT1 substrate RelB and accumulation of BCL10 were analyzed of HBL1, OCI-Ly10, TMD8 and OCI-Ly3 cells (2.5 × 10^5^/ml) with increasing concentrations of Ibrutinib **B.** or S-Mepazine **C.** for 18h was as in A. Western Blots detect decrease of cleaved RelBΔ and accumulation of BCL10 full-length protein upon treatment. **C.** Accumulation of full length BCL10 was directly compared after treatment of ABC DLBCL cells with increasing doses of S-Mepazine alone or in combination with 0.5 nM Ibrutinib for 18 h. All Western Blots show a representative experiment from at least three independent experiments.

We asked if decreased MALT1 activity also coincides with a reduction of MALT1 substrate cleavage. For this, ABC DLBCL cells were incubated with Ibrutinib (5 nM) and S-Mepazine (10 μM) and cleavage of the MALT1 substrates RelB and BCL10 was detected by Western Blot (Figure 2A). Both inhibitors prevented RelB and BCL10 cleavage in HBL1, TMD8 and OCI-Ly10 cells, but only the MALT1 inhibitor S-Mepazine was able to effectively inhibited MALT1 substrate cleavage in OCI-Ly3 cells. MALT1 cleaves BCL10 at the very C-terminus and as observed in previous publications inhibition of MALT1 promoted strong accumulation of full-length BCL10 in ABC DLBCL cells [16, 17]. Accumulation of full-length BCL10 upon MALT1 inhibition was best detected with an antibody (EP606Y) directed against the BCL10 C-terminus that does not recognize cleaved BCL10Δ (Figure 2A). Next, ABC DLBCL cells were incubated in the presence of Ibrutinib (0.5-5 nM) and MALT1 inhibition was monitored by detecting accumulation of uncleaved BCL10 and decline of the RelB cleavage product (RelBΔ) (Figure 2B). Congruent with the direct effects on MALT1 activity, BTK inhibition by Ibrutinib inhibited cellular substrate cleavage only in HBL1, TMD8 and OCI-Ly10 cells in a dose dependent manner. S-Mepazine was effectively inhibiting RelB and BCL10 cleavage in all cells independent of the oncogenic event at concentrations between 0.5-10 μM (Figure 2C). We assessed combinatorial effects on MALT1 substrate cleavage and we chose BCL10 accumulation, because the increase in the uncleaved form can be reliably monitored in all cells (see Figure 2A). Cells were treated with increasing concentrations of S-Mepazine in the absence or presence of 0.5 nM Ibrutinib. Indeed, combinatorial treatment led to augmented inhibition of MALT1-dependent BCL10 cleavage in HBL1, OCI-Ly10 and TMD8 cells, but not in OCI-Ly3 cells (Figure 2D). Taken together, the data show that combination of BTK and MALT1 inhibitors exerts additive effects on MALT1 inhibition in CD79 mutant cells.

### Augmented depletion of NF-κB dependent survival factors in CD79 mutant cells by BTK and MALT1 inhibition

**Figure 3:**
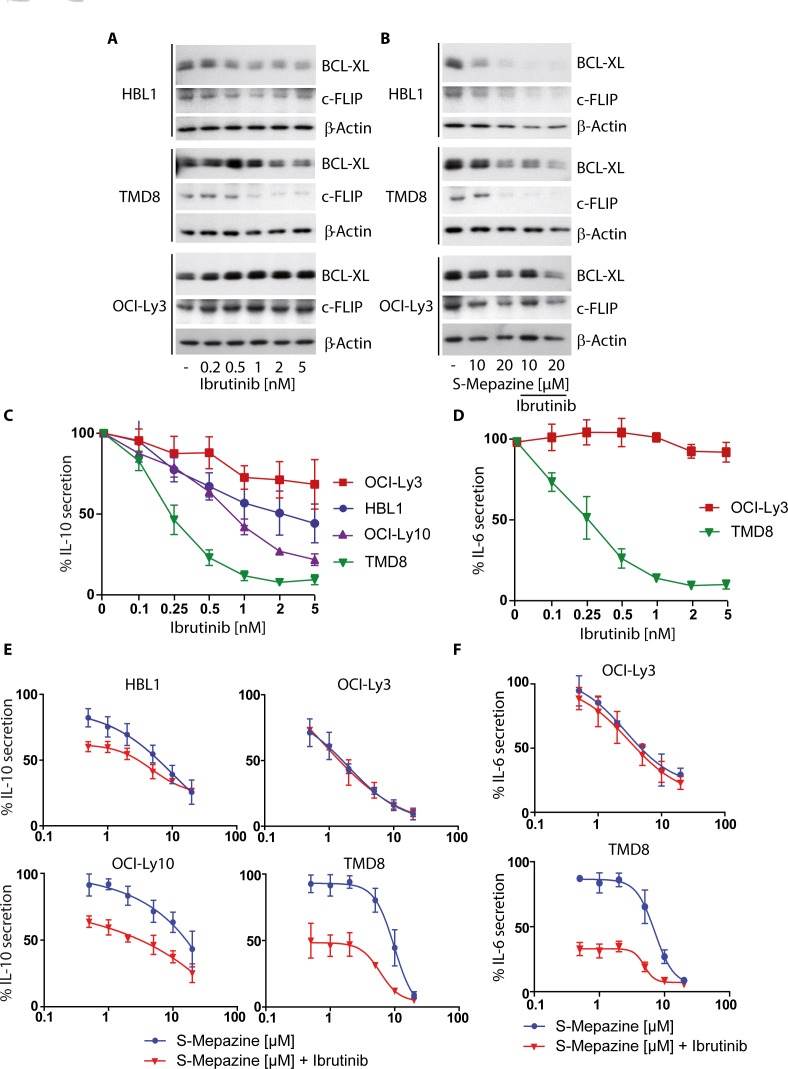
Additive reduction of NF-κB regulated apoptosis factors and cytokines in CD79 mutant ABC DLBCL cells **A.** BCL_XL_ and c-FLIP protein levels were detected via Western Blot after incubation of HBL1, TMD8 and OCI-Ly3 cells (2.5 × 10^5^/ml) with increasing concentrations of Ibrutinib for 24 h. **B.** BCL_XL_ and c-FLIP protein levels were detected via Western Blot after incubation of HBL1, TMD8 and OCI-Ly3 cells (2.5 × 10^5^/ml) with S-Mepazine (10 and 20 μM) with or without Ibrutinib co-treatment (2 nM for HBL1 and OCI-Ly3 cells; 1 nM for TMD8 cells). Blots in A and B show a representative of at least three independent experiments. **C.** and **D.** ABC DLBCL cells (2.5 × 10^5^/ml) were incubated with Ibrutinib for 18 h and secreted IL-10 **C.** and IL-6 **D.** amounts were detected by ELISA. **E.** and **F.** ABC DLBCL cells (2.5 × 10^5^/ml) were incubated with increasing concentrations of S-Mepazine alone or in combination with Ibrutinib and secreted IL-6 and IL-10 amounts were measured by ELISA after 18 h. Based on Ibrutinib single agent treatment, HBL1, OCI-Ly10 and OCI-Ly3 cells were treated with 0.5 nM Ibrutinib and TMD8 cells with 0.25 nM Ibrutinib. Data in C-F show the mean of three independent experiments ± SD.

The survival of ABC DLBCL cells is strongly dependent on constitutive NF-κB activation that promotes protection from apoptosis. The anti-apoptotic proteins BCL_XL_ and c-FLIP are induced via NF-κB-dependent gene expression and are required to maintain survival of ABC DLBCL cells. To measure the effects of combinatorial S-Mepazine and Ibrutinib application we detected BCL_XL_ and c-FLIP proteins in HBL1, TMD8 and OCI-Ly3 cells (Figure 3A and 3B). Upon Ibrutinib treatment alone, BCL_XL_ and c-FLIP amounts were reduced in HBL1 and TMD8 cells, but not in OCI-Ly3 cells (Figure 3A). S-Mepazine caused reduced expression of both survival factors in all three ABC DLBCL cells (Figure 3B). Whereas a combination of both compounds resulted in an additive reduction of both proteins in CD79 mutant HBL1 and TMD8 cells, Ibrutinib did not further reduce the S-Mepazine triggered decreases of BCL_xL_ and c-FLIP in CARMA1 mutant OCI-Ly3 cells.

NF-κB-dependent gene expression of IL-6 and IL-10 is important for ABC DLBCL cells, because they act on the cell survival and growth in an autocrine fashion [21]. We determined IL-10 secretion after Ibrutinib treatment in HBL1, TMD8, OCI-Ly10, and OCI-Ly3 cells (Figure 3C). Strong IL-6 expression was only detected in the ABC DLBCL cell lines TMD8 and OCI-Ly3 [19] and therefore we also determined the impact of Ibrutinib on IL-6 in these two cells (Figure 3D). Again, Ibrutinib was only able to impair IL-6 or IL-10 expression in ABC DLBCL cells that express CD79 mutants, but not in CARMA1 L244P expressing OCI-Ly3 cells. In contrast, S-Mepazine alone was able to suppress IL-6 and IL-10 expression in all ABC DLBCL cell lines, but the combination of both BTK and MALT1 inhibitors was severely augmenting the inhibitory effects on production of both interleukins in CD79 mutant ABC DLBCL cells (Figure 3E and 3F). Thus, the combination of BTK and MALT1 inhibitors apparently additively increases the efficacy over single agent treatment to suppress expression of NF-κB dependent pro-survival proteins in ABC DLBCL cells.

### Combinatorial S-Mepazine and Ibrutinib treatment enhanced toxicity in CD79 mutant DLBCL cells

**Figure 4:**
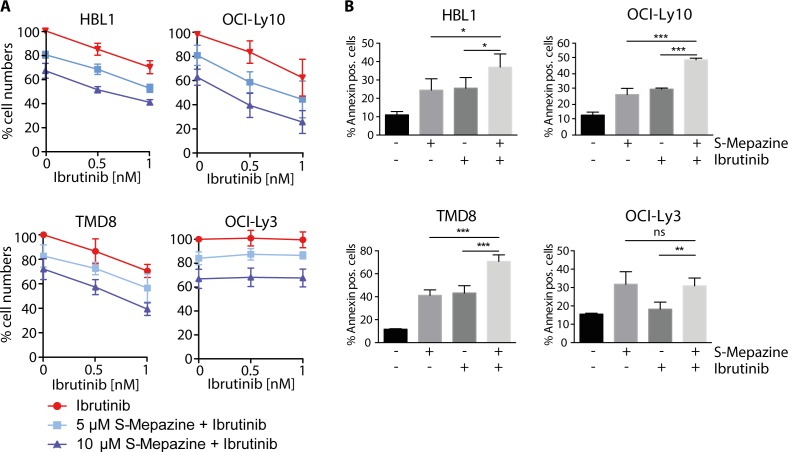
Augmented toxicity of Ibrutinib and S-Mepazine treatment in ABC DLBCL cells **A.** Detection of viable ABC DLBCL cells after 4 days of S-Mepazine (10 and 20 μM) or Ibrutinib (0.5 and 1 nM) alone or in combination of both drugs at the respective concentrations. Viable cells are given in relation to DMSO treated control. **B.** ABC DLBCL cells (2.5 × 10^5^/ml) were treated with DMSO, S-Mepazine (10 μM), Ibrutinib (1 nM) or in combination and the percentage of the Annexin-V-FITC positive and YO-PRO-3 negative population was determined after day 3 via FACS analysis. All data represent the mean of three independent experiments ± SD. Significance was calculated by Student's t-test (*p ≤ 0.05; **p ≤ 0.01; ***p ≤ 0.001).

NF-κB pro-survival prevents cell death in ABC DLBCL cells and mono-treatment with BTK and MALT1 inhibitors exerts toxic effects [4, 18, 19]. Therefore, we monitored the number of viable cells in response to Ibrutinib and S-Mepazine treatment alone or in combination (Figure 4A). Ibrutinib at 0.5 and 1 nM was not toxic to OCI-Ly3 cells, but led to a dose dependent decline of viable cells in all other ABC DLBCL cell lines. All ABC DLBCL cells were sensitive to MALT1 inhibitor S-Mepazine at 5 and 10 μM. Correlating with the effects on cellular MALT1 activity and expression of pro-survival proteins upon combinatorial incubation, the toxicity was further increased by the parallel incubation of Ibrutinib and S-Mepazine in CD79 mutant cells. Again, Ibrutinib was not further augmenting toxicity of S-Mepazine in OCI-Ly3 cells.

We also determined induction of apoptosis in HBL1, TMD8, OCI-Ly10 and OCI-Ly3 cells by determining AnnexinV positive cells 3 days after single agent or combinatorial treatment (Figure 4B). Whereas Ibrutinib (1 nM) and S-Mepazine (10 μM) alone weakly induced apoptosis in GroΔrutinib sensitive ABC DLBCL cells, the combination of both drugs significantly enhanced apoptosis when compared to single agent treatment. As expected, apoptosis in OCI-Ly3 cells was increased upon S-Mepazine administration, but the cells did not react to Ibrutinib treatment. Thus, the data underscore that combination of BTK and MALT1 inhibitors can enhance toxicity in CD79 mutant ABC DLBCL cells.

**Figure 5:**
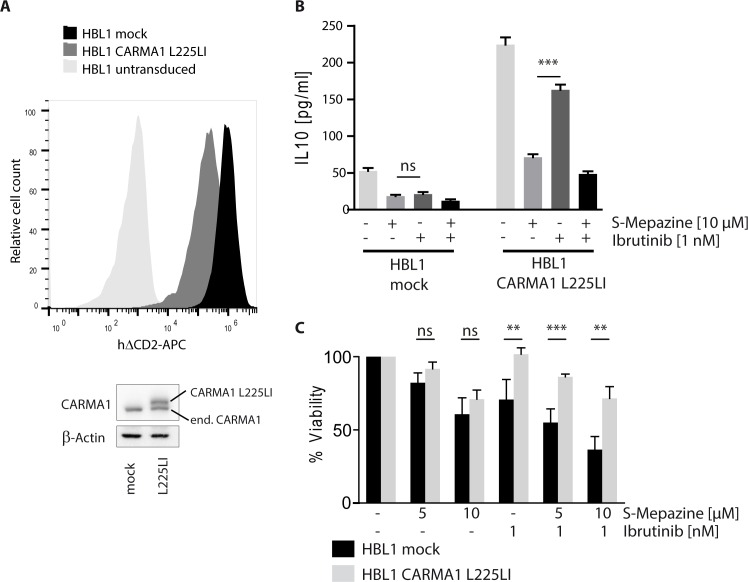
Sensitivity of Ibrutinib and S-Mepazine treatment relies on oncogenic background of DLBCL cells **A.** HBL1 cells were lentivirally infected either with mock or with a construct encoding oncogenic CARMA1 mutant L225LI with high efficiency. ΔCD2 co-expression of transduced cells was measured in FACS. Cell lysates were analyzed in Western Blot for expression of CARMA1 protein (endogenous and exogenous). **B.** Mock or CARMA1 L225LI transduced HBL1 cells (2.5 × 10^5^/ml) were treated with DMSO, S-Mepazine or Ibrutinib or the combination as indicated. Secreted IL-10 amounts were measured by ELISA after 18 h. **C.** HBL1 cells were transduced with mock or CARMA1 L225LI construct and treated with S-Mepazine alone or in combination with Ibrutinib. Detection of viable HBL1 cells after 4 days of S-Mepazine (5 and 10 μM) or Ibrutinib (1 nM) alone or in combination of both drugs are given in relation to DMSO treated control. Data in B and C show the mean of three independent experiments ± SD. Significance was calculated by Student's *t*-test (**p ≤ 0.01; ***p ≤ 0.001).

Binding to phenothiazines is impaired by the mutation E397A in the allosteric binding site of MALT1 [20]. Congruently, we have been able to show that expression of MALT1 E397A rendered HBL1 cells resistant to MALT1 inhibitor treatment, verifying that Mepazine is exerting toxic effects by acting on MALT1 [20]. Despite the fact that OCI-Ly3 cells that carry oncogenic CARMA1 mutation are resistant to Ibrutinib treatment, we wanted to verify that indeed the oncogenic status of the ABC DLBCL cells independent of their origin determines drug sensitivity. For this, we expressed the activating CARMA1 mutant L225LI in HBL1 cells to potentially render the cells independent of BCR upstream signaling [5]. HBL1 cells were transduced with high efficiency (>95%) upon lentiviral infection as evident from co-expression of the surface marker ΔCD2 (Figure 5A). Strep-Flag-CARMA1 L225LI was expressed approximately at endogenous levels of CARMA1 in HBL1 cells. We determined effects of BTK and MALT1 inhibition by measuring IL-10 production (Figure 5B). IL-10 amounts in mock infected HBL1 cells were decreased after Ibrutinib and S-Mepazine treatment either alone or in combination. Expression of oncogenic CARMA1 L225LI further boosted IL-10 secretion. Despite the strong induction, IL-10 expression in CARMA1 L225LI HBL1 cells was still severely reduced by MALT1 inhibitor S-Mepazine, but much more resistant to incubation of the BTK inhibitor Ibrutinib.

Finally, we compared toxic effects of Ibrutinib and S-Mepazine in mock and CARMA1 L225LI expressing HBL1 cells (Figure 5C). Whereas mock and CARMA1 L225LI infected cells were still sensitive to MALT1 inhibitor S-Mepazine, CARMA1 L225LI conferred resistance to BTK inhibitor Ibrutinib. Additionally, CARMA1 L225LI abolished augmented killing by combinatorial treatment of Ibrutinib and S-Mepazine. Thus, the data demonstrate that sensitivity to the compounds as well as combinatorial treatment is indeed relying on the oncogenic background of the DLBCL cells.

## DISCUSSION

Resistance mechanisms are a major obstacle for precision therapy and target directed approaches for cancer therapy. Combinatorial treatment protocols with different drugs that target cancer at various stages of one or more oncogenic pathways are thought to reduce the chances of drug resistance over single agent therapy. Promising preclinical and clinical data have demonstrated high potency of BTK inhibitor Ibrutinib for the treatment of various B cell malignancies, such as MCL, CLL and ABC DLBCL [6]. However, the advent of Ibrutinib resistances in CLL and MCL stressed the limitations of BTK monotherapy [13, 15]. Especially the frequent BTK mutation C481S that hinders binding of Ibrutinib is leading to relapse in many lymphoma patients. Recently, a phase 1/2 clinical trial demonstrated that Ibrutinib produces partial or complete responses in ABC DLBCL patients that rely on chronic BCR signaling, but is ineffective in patients carrying activating CARMA1/CARD11 mutations [11]. Even though so far no BTK resistant mutations have been described in ABC DLBCL, clinical responses are apparently not long-lasting, stressing the necessity to develop drug combinations (reviewed in [12]). Our results suggest that combinatorial BTK and MALT1 inhibitor treatment may enhance efficacy, but can also reduce the chances of resistances that are acquired either by mutations of the drug targets or indirectly in downstream components such as CARMA1.

Previous studies have largely focused on combining BTK inhibitors with inhibitors and candidate drugs that target parallel oncogenic pathways. In ABC DLBCL cells, high degree of cooperativity has been reported for combining Ibrutinib with inhibitors targeting the parallel SYK-PI3K-AKT-mTORC pathway that emanates upstream from BTK [4, 23, 24]. Synergistic effects were also obtained by combining BTK with either BCL2 inhibitors that enhance apoptosis or lenalidomide that increases toxic interferon responses in ABC DLBCL [10, 24]. Even though compounds targeting parallel oncogenic pathways exert a strong synergistic response together with Ibrutinib, this approach may not be sufficient to prevent the development of Ibrutinib resistances by BTK or downstream mutations. Therefore, we took an alternative approach and explored the combinatorial application of the irreversible BTK inhibitor Ibrutinib with the allosteric MALT1 inhibitor S-Mepazine, because both compounds act at different stages of the pathological BCR-NF-κB signaling axis. Indeed, by correlating inhibitor sensitivity to oncogenic alterations in well-defined cell lines we could confirm that BTK is an upstream regulator of MALT1 activity in CD79 mutant, but not in CARMA1 mutant DLBCL cells. In contrast, MALT1 inhibitor S-Mepazine was still effective in CARMA1 mutant DLBCL that are resistant to Ibrutinib. In cells that were sensitive to BTK inhibition, the combination of both inhibitors resulted in augmented MALT1 inhibition and concomitantly decreased expression of BCR-NF-κB regulated proteins and induced toxicity at lower concentrations of the individual compounds. Specificity of the inhibitors was confirmed by expressing oncogenic CARMA1 L225LI or S-Mepazine binding mutant MALT1 E397A that confers resistance to BTK or MALT1 inhibition, respectively [20].

In contrast to the synergistic effects observed for instance by the combination of BTK and PI3K-AKT inhibitors [24], BTK and MALT1 co-treatment yielded additive effects on MALT1 activity and killing of CD79 mutant ABC DLBCL cells. It confirms that both inhibitors are primarily targeting pathological BCR-NF-κB signaling. Nevertheless, inhibiting different steps of the same oncogenic pathway may enhance efficacy of treatment and significantly reduce the chances to develop Ibrutinib resistances compared to single agent therapy. In addition, lowering the doses of individual pharmaceuticals may decrease toxic adverse effects, as some side effects have already been observed in clinical trials with Ibrutinib [25]. Phenothiazines like Thioridazine or Mepazine have been used since the 1950ies to treat anti-psychotic disorders. Thioridazine is still an approved drug opening up the option of drug repositioning and repurposing trials in relapsed/refractory ABC DLBCL potentially in combination with Ibrutinib. By enhancing lymphoma killing and preventing the development of resistances, combinatorial regimes may result in more long-lasting responses and hopefully avoid the necessity for lifelong treatment with the inhibitors. Furthermore, BTK inhibition was also shown to impact on CARMA1-NF-κB signaling in a subset of MCLs [26]. It will be interesting to unravel whether MALT1 may be active in other Ibrutinib sensitive lymphomas such as CLL or MCL and to determine whether combinatorial BTK and MALT1 inhibition may potentially hold benefits for lymphoma patients beyond ABC DLBCL.

## MATERIALS AND METHODS

### Cell culture and reagents

DLBCL cell lines were cultured in RPMI 1640 medium (Invitrogen) supplemented with 20 % FBS and 100 U/ml penicillin/streptomycin. OCI-Ly10 cell line was cultured in IMDM medium (Invitrogen) with 20 % human plasma, penicillin/streptomycin and 50 μM β-mercaptoethanol. Jurkat T cells were cultured like DLBCL cells with 10 % FCS. S-Mepazine HCl (Kalexsyn) and Ibrutinib (Selleck Chem) were solved in DMSO and used as indicated. For detection of RelB cleavage fragment proteasome inhibitor MG132 (Merck; 10 μM) was incubated 1h prior to lysis. Antibodies for Western Blot were BCL10 (Abcam EP606Y and Santa Cruz H197), RelB (Cell signaling, C1E4), BCL_XL_ (Cell Signaling), MALT1 (H300, Santa Cruz), c-FLIP (Alexis Biochemicals) and β-Actin (I-19, Santa Cruz). IL-6 and IL-10 ELISAs (Immunotools) were performed according to the manufacturer's protocols.

### Apoptosis and cell count assays

Viability of DLBCL cell lines was analyzed using a Vi-Cell cell counter (Beckman Coulter) after four days of S-Mepazine and/or Ibrutinib treatment in comparison to DMSO-treated cells. Apoptosis rates of the cells was determined accordingly after 3 days of compound treatment calculating the percentage of Annexin V-FITC positive and YO-PRO-3 negative stained cells (BD) by FACS (Attune, Life technologies).

### Lentiviral transduction

For lentiviral infection of HBL1 cells with the construct for CARMA1 L225LI HEK293T cells were transfected each with pMD2.G and psPAX2 lentiviral packaging plasmids using X-tremeGENE HP Transfection Reagent (Roche). As a transfer vector we used 2 μg of a pHAGE construct containing hΔCD2 followed by the T2A and CARMA1 L225LI sequence. After 3 days the virus was concentrated in Amicon ultra 100K Centrifugal filters (Millipore). 200 000 HBL1 cells were transduced via spin infection for 1 h and 720 g at room temperature. For increased efficiency of infection we added Polybrene to a concentratin of 8 μg/ml followed by incubation overnight. Infection rates of HBL1 cells were monitored by anti hΔCD2-APC (eBioscience) staining in flow cytometry.

### MALT1 ELASA (enzyme-linked activity-sorbent assay)

Activity of MALT1 was determined as described earlier [22]. To this end DLBCL cell lines (5 × 10^5^/ml per sample) were treated with different concentrations of Ibrutinib and S-Mepazine for 18 h and lysed in 130 μl co-IP buffer (25 mM hepes pH 7.5, 150 mM NaCl, 10% glycerol, 0.2% NP-40, 1mM DTT, 10 mM sodium fluoride, 8 mM β-glycerophosphate and 300 μM sodium vanadate) w/o protease inhibitors. After centrifugation and removal of 10 μl input control, extract was incubated with 0.1 μM biotinylated ABP (activity-based probe) at RT for 1 h to form active MALT1-ABP complexes. Extract was transferred to streptavidin-coated plates (Thermo Scientific) and incubated o/n at 4°C. Plates were washed with PBS-T (0.05% Tween), blocked with 2% BSA and incubated with primary MALT1 antibody (2494; Cell Signaling) for 1h at RT. After further washing, HRP-conjugated secondary antibodies were added for 1 h, washed and TMB substrate was incubated for approximately 15 min. Reaction was stopped with 1M H_2_PO_4_ and luminescence was analyzed in a Biotek reader at 450 nm (background 570 nm).
